# Modulation of the porcine intestinal microbiota in the course of *Ascaris suum* infection

**DOI:** 10.1186/s13071-022-05535-w

**Published:** 2022-11-17

**Authors:** Andrea Springer, Liane Wagner, Sarina Koehler, Stefanie Klinger, Gerhard Breves, Dagmar A. Brüggemann, Christina Strube

**Affiliations:** 1 Institute for Parasitology, Centre for Infection Medicine, University of Veterinary Medicine Hannover, Buenteweg 17, 30559 Hanover, Germany; 2 Department of Safety and Quality of Meat, Max Rubner-Institut, Federal Research Institute of Nutrition and Food, E.-C.-Baumann-Straße 20, 95326 Kulmbach, Germany; 3 Institute for Physiology and Cell Biology, University of Veterinary Medicine Hannover, Bischofsholer Damm 15, 30173 Hanover, Germany

**Keywords:** Ascarids, Roundworms, Trickle infection, Intestinal microbiota, Microbiome, Network analysis, Short-chain fatty acids

## Abstract

**Background:**

The porcine roundworm *Ascaris suum* impairs feed conversion and weight gain, but its effects on intestinal microbiota remain largely unexplored.

**Methods:**

Modulation of the intestinal microbiota was assessed in pigs that were infected once with 10,000 *A. suum* eggs and pigs that received a trickle infection (1000 eggs/day over 10 days), compared with a non-infected control group. Six pigs each were sacrificed per group at days 21, 35 and 49 post-infection (p.i.). Faecal samples taken weekly until slaughter and ingesta samples from different intestinal compartments were subjected to next-generation sequencing of the bacterial 16S rRNA gene.

**Results:**

The results revealed marked differences between the single- and the trickle-infected group. Single infection caused a remarkable but transient decrease in microbial diversity in the caecum, which was not observed in the trickle-infected group. However, an increase in short-chain fatty acid-producing genera in the caecum on day 21 p.i., which shifted to a decrease on day 35 p.i., was common to both groups, possibly related to changes in excretory–secretory products following the parasite’s final moult. Faecal microbial interaction networks were more similar between the single-infected and control group than the trickle-infected group. In addition, a lower degree of similarity over time indicated that *A. suum* trickle infection prevented microbiota stabilization.

**Conclusions:**

These different patterns may have important implications regarding the comparability of experimental infections with natural scenarios characterized by continuous exposure, and should be confirmed by further studies.

**Graphical Abstract:**

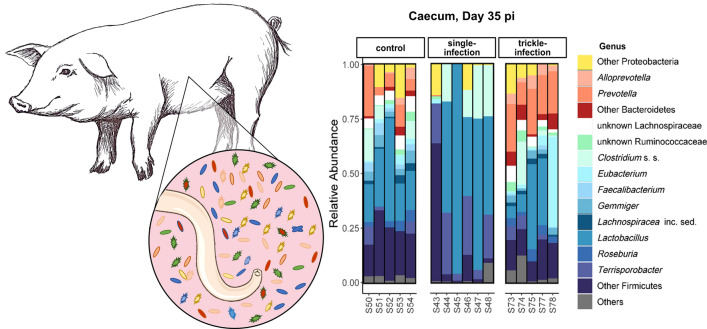

**Supplementary Information:**

The online version contains supplementary material available at 10.1186/s13071-022-05535-w.

## Background

Gastrointestinal parasites share their habitat with a diverse community of bacteria, the gastrointestinal microbiota, which plays a profound role in host metabolism, physiological development, immune regulation and overall health [1]. Consequently, multilateral relationships exist between intestinal parasites, the microbiota and host cells, shaping the physiological consequences of infection [2].

The roundworm *Ascaris suum* is one of the economically most important endoparasites in commercial pig farming, and is closely related to or even conspecific with the human roundworm *Ascaris lumbricoides* [3], which affects approximately 800 million people worldwide [4]. After oral infection with embryonated eggs, *A. suum* larvae penetrate the caecal or colonic mucosa, enter the bloodstream and undergo a body migration via the liver and the lungs, before reaching the small intestine approximately 8–10 days post-infection (p.i.) [5]. In the jejunum, they continue their development; however, a large proportion of the fourth-stage larvae (L4) are eliminated between days 17 and 21 p.i., irrespective of the infection dose. This so-called self-cure phenomenon is associated with an increase in mucosal eosinophils, macrophages and T-cells and decreased gastrointestinal transit time [6]. The remaining larvae undergo a final moult between days 21 and 29 p.i. [7], and the onset of patency occurs approximately 6–8 weeks p.i. [8].

*Ascaris suum*-related production losses result from reduced weight gain and feed conversion efficiency, in addition to condemnation of livers showing characteristic lesions due to the parasite’s body migration (so-called milk spots), costs for helminth control, and indirect, immunomodulatory effects of the parasite [9]. Although it has been difficult to demonstrate consistent changes in feed utilization and weight development of infected versus non-infected pigs, several experimental and field studies have reported significant differences (reviewed in [9]). For example, a 12% reduction in live weight gain was found in *A. suum*-infected versus anthelminthic-treated pigs in an outdoor system [10]. Reduced feed utilization efficiency is presumably due to a parasite-induced reduction in sodium-coupled intestinal glucose resorption [11–14]; however, alterations of the intestinal microbiota might also play a role. It has been suggested that *A. suum* actively manipulates its microbial environment by the release of potent antimicrobial proteins and peptides, thereby creating a favourable metabolic environment and immunoregulatory state [2, 15]. Furthermore, changes in intestinal physiology during infection, such as increased or disrupted mucus production, are likely to affect microbial populations in the gut, as suggested by an increase in *Mucispirillum* spp. in *Trichuris suis*-infected pigs [16].

Indeed, changes in the porcine intestinal microbiota have been observed during the early phase (day 14 p.i.) of *A. suum* infections [17, 18], as well as on day 54 p.i. [19]. The results were somewhat incongruent, since increased colonic microbial diversity was noted in the early phase [17], but a decrease was seen in the chronic phase of infection [19]. Furthermore, since *A. suum* resides in the small intestine, effects in other intestinal compartments may differ from those observed in the colon [18]. Therefore, the present study aimed to comprehensively characterize microbial changes during the course of *A. suum* infection by analysing faecal samples taken in weekly intervals until day 49 p.i., and ingesta samples from the jejunum, ileum, caecum and colon taken at days 21, 35 and 49 p.i. Furthermore, continuous, low-dose infections are common under natural conditions, and may have different consequences from those of the single-dose experimental infections mentioned above [20]. The current study therefore compared single-dose-infected pigs and pigs trickle-infected over the course of 10 days to an uninfected control group.

## Methods

### Experimental *A. suum* infections and sample collection

*Ascaris suum* eggs were obtained from adult worms collected at an abattoir and allowed to embryonate at 25 °C for 2 months. Embryonated eggs were subsequently kept at 4 °C until oral infection of pigs.

Infection experiments were performed in a project on intestinal nutrient transport [13]. Fifty-four helminth-free hybrid German Landrace pigs weighing approximately 10 kg and 5 weeks of age were acquired from the *A. suum*-free Ruthe Research and Education Farm of the University of Veterinary Medicine Hannover, Germany. Faecal samples were taken rectally on the day of the pigs’ arrival and subjected to coproscopic analysis to verify they were helminth-free. The pigs were divided into three groups, which were kept in rooms with concrete flooring on straw bedding. They received a standard pig diet and water ad libitum (Deuka Ferkelstarter Primo, Deutsche Tiernahrung Cremer, Düsseldorf, Germany). After a minimum adaptation period of 1 week, 18 pigs each received either a single oral infection of 10,000 embryonated *A. suum* eggs or a trickle infection of 1000 eggs/day for 10 days (10,000 eggs in total), while a further 18 pigs served as a non-infected control. To ensure that all pigs had received the same overall infection dose by the time of investigation, the trickle infection was not spread over more than 10 days. Pigs were kept until slaughter on days 21, 35 and 49 p.i. (*N* = 6 per group and time point), respectively. Serum samples were taken weekly from each pig and subjected to anti-*Ascaris* antibody detection to verify successful infection [human anti-*Ascaris lumbricoides* immunoglobulin G (IgG) enzyme-linked immunosorbent assay (ELISA), Abcam (Netherlands) B.V., Amsterdam, Netherlands]. In addition, macroscopically visible worms were collected from the intestines upon slaughter. To monitor weight gain, pigs were weighed upon arrival and on the day of necropsy.

**Fig. 1:**
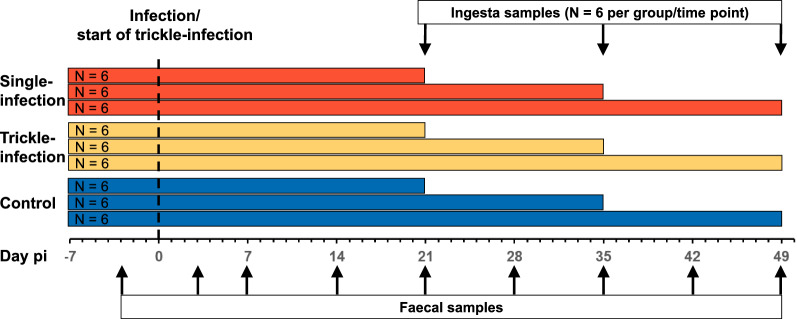
Sampling strategy to assess the effect of *A. suum* infection on the gastrointestinal microbiota of pigs

Faecal samples for microbiota analysis were taken rectally 3 days prior to infection and on days 3, 7, 14, 21, 28, 35, 42 and 49 p.i., and stored at −80 °C until further processing. Intestinal contents were sampled with sterile tools from the proximal jejunum (approximately 3 m distal to the pylorus), medial jejunum (approximately 6–8 m distal to the pylorus), ileum, caecum and colon ascendens (second coil) and frozen immediately in liquid nitrogen, before storage at −80 °C. The experimental setup and sampling scheme is visualized in Fig. 1.

### DNA isolation, polymerase chain reaction (PCR) and sequence processing

DNA isolation from faecal samples was performed with the DNeasy^®^ PowerLyzer^®^ PowerSoil^®^ kit (Qiagen, Hilden, Germany) with a homogenization step of 2 × 45 s at 5000 rpm in a Precellys^®^ 24 tissue homogenizer (Peqlab Biotechnologie GmbH, Erlangen, Germany). The procedure followed the manufacturer’s instructions, except that centrifugation time at step 10 was increased to 2 min. For ingesta samples, the QIAamp^®^ BiOstic^®^ Bacteremia DNA Kit (Qiagen, Hilden, Germany) was used, as this kit has been shown to increase bacterial DNA yield in low-biomass (i.e. small intestine) samples [21]. All steps followed the manufacturer’s instructions, except that bead-beating was performed with the Precellys^®^ 24 as described above, and centrifugation time at step 7 was increased to 2 min. Eluted DNA was stored at −20 °C until shipment to a commercial laboratory (Microsynth AG, Balgach, Switzerland).

Illumina MiSeq sequencing, sequence processing, operational taxonomic unit (OTU) clustering and taxonomic assignment were performed by Microsynth AG (Balgach, Switzerland), according to Microsynth’s standard operating procedures. After dilution of the samples to 2.5 ng DNA/µl, two-step Nextera PCR libraries of the V3–V4 region of the bacterial 16S rRNA gene were created using KAPA HiFi Mastermix (Roche Sequencing and Life Science, Indianapolis, IN, USA). The first-step PCR with primers 341F and 805R (4 µm each) [22] and the second-step PCR with index primers (4 µm each, including Illumina adapters) comprised 20 and 10 cycles of the following thermoprofile, respectively: 95 °C 3 min; 20/10 × 98 °C 20 s, 56 °C 30 s, 72 °C 30 s; 72 °C 5 min. The second-step PCR products were bead-purified, quantified with fluorescence spectroscopy and pooled in equimolar amounts. The Illumina MiSeq platform and a v3 600-cycle kit (both Illumina Inc., San Diego, CA, USA) were used for sequencing. The resulting paired-end reads which passed Illumina’s chastity filter were subjected to de-multiplexing and adaptor trimming using Illumina’s real-time analysis software included in the MiSeq Reporter software v. 2.6 (no further refinement or selection). Read quality was checked with FastQC v. 0.11.8 (Babraham Institute, Cambridge, UK), and the locus-specific V3–V4 primers were trimmed with Cutadapt v. 2.8 [23], discarding reads if primer trimming failed. Further, the reads were trimmed from the original 300 base pairs (bp) to 250 bp from the 3′ end to reduce sequencing noise. Trimmed forward and reverse reads were merged in silico, considering a minimum overlap of 15 bases using USEARCH v. 11.0.667 [24]. Merged sequences were quality-filtered, allowing a maximum of one expected error per merged read. Reads with ambiguous bases and outliers regarding amplicon size were discarded. The remaining reads were denoised via the UNOISE algorithm [25] to form OTUs, discarding singletons and chimeras. The resulting OTU abundance table was filtered for possible bleed-in contaminations using the UNCROSS algorithm [26]. OTUs were compared against the reference sequences of the Ribosomal Database Project (RDP) 16S database [27], and taxonomies were predicted considering a minimum confidence threshold of 0.5 using the SINTAX algorithm implemented in USEARCH [28]. OTU clustering and taxonomic assignment were performed separately for faecal samples and for small intestine (jejunum, ileum) and large intestine (colon, caecum) samples, resulting in three separate datasets.

### Statistical analyses

Statistical analyses were conducted in R v. 4.1.0 [29], using the packages phyloseq [30], vegan [31], DESeq2 [32] and lmerTest [33]. The average daily weight gain until day 21, day 35 and day 49 p.i. was compared between the experimental groups by Kruskal–Wallis tests, followed by Dunn’s post hoc tests.

Prior to calculating microbial alpha and beta diversity indices, the faecal sample dataset was rarefied to 3000 reads/sample and the ingesta datasets to 8000 reads/sample. Rarefaction curves were inspected to verify that the level of rarefaction adequately captured the phylogenetic diversity.

To assess microbial diversity within each sample (i.e. alpha diversity), the number of observed OTUs and the Chao1, Shannon and inverse Simpson indices were calculated, with the latter two taking not only the absence/presence, but also the abundance of OTUs into account [34]. For faecal samples, the influence of day p.i., experimental group and their interaction on alpha diversity was assessed by generalized linear mixed models (GLMMs), with animal ID as a random factor due to repeated sampling of individuals. A Poisson error structure with identity link was used to model observed OTUs, while a gamma distribution with identity link proved more appropriate for Chao1 and Shannon indices, and a gamma distribution with log link for the inverse Simpson index. Full models were compared with null models containing only the random factor in a maximum likelihood test, and the distribution of model residuals as well as residuals against fitted values were inspected graphically to assess model fit. Alpha diversity of ingesta samples was compared between experimental groups for each intestinal segment and day p.i. using Kruskal–Wallis tests with false discovery rate (FDR) correction of *P*-values, followed by Dunn’s post hoc test as appropriate.

To assess differences in microbiota composition (i.e. beta diversity), Jensen–Shannon distances (JSD) were calculated and analysed by permutational analysis of variance (PERMANOVA, 10,000 permutations). For faecal samples, the initial PERMANOVA included the predictors experimental group, day p.i. and their interaction, and was stratified by animal ID to account for repeated sampling. Because all predictors were significant in the initial model, separate PERMANOVAs were subsequently calculated for each infection group versus the control group, and for each sampling day to investigate differences in detail. Likewise, separate PERMANOVAs were calculated for each infection group versus the control group and each day p.i. regarding the ingesta samples.

The relative abundance of microbial phyla and genera was compared among the three groups at each day p.i. using Kruskal–Wallis tests with FDR correction of *P*-values. Dunn’s post hoc tests were conducted if corrected *P*-values were ≤ 0.05. To further analyse differences in the abundance of specific microbial taxa at the species level, DESeq2 differential expression analyses were conducted using the non-rarefied datasets, as DESeq2 performs an internal normalization [32].

### Microbial network analysis

Microbial association networks were constructed based on faecal samples, as the number of only six pigs slaughtered per group at each time point did not allow network construction for ingesta samples. Networks were constructed for days −3, 14, 21 and 35 p.i. via the R package “NetCoMi” [35] using the SPIEC-EASI (Sparse Inverse Covariance Estimation for Ecological Association Inference) association measure, which takes the compositional structure of amplicon-based datasets into account [36]. This was not possible for day 49 due to the smaller sample size per group at this time point. For network construction, OTUs present in less than 20% of all faecal samples were excluded, but no prior rarefaction was performed, as normalization is included in the “spiec.easi” function. Networks were calculated separately for each group and day p.i. The greedy modularity algorithm was used for network clustering [37]. Normalized centrality measures (degree, betweenness, closeness and eigenvector centrality) were calculated. “Hub” OTUs, i.e. influential OTUs in the networks, were defined as having an eigenvector centrality value above the 95% quantile of the empirical eigenvector centrality distribution [35]. Eigenvector centrality is a measure of influence in a network, which takes not only the number of connections of a node (i.e. the degree) into account, but also the degree of its neighbours.

## Results

All control animals remained *Ascaris*-seronegative throughout the study period. Of the 36 animals in the infection groups, five were excluded from the study (two from the single- and three from the trickle-infected group) as they did not seroconvert until slaughter and had no visible worms in the intestines. The average daily weight gain ranged from 0.28 to 0.5 kg for pigs slaughtered on day 21 p.i., from 0.30 to 0.47 kg for those slaughtered on day 35 p.i. and from 0.32 to 0.60 kg for those slaughtered on day 49 p.i. A significant difference in daily weight gain between the experimental groups was observed only on day 49 p.i. (Kruskal–Wallis *χ*^2^ = 7.5, *df* = 2, *P* = 0.024), with significantly higher daily weight gain in the trickle-infected than in the single-infected and the control groups (Dunn’s all-pairs test, Bonferroni–Holm-adjusted *P* = 0.048 for both comparisons).

###  Faecal samples

#### Alpha and beta diversity

The quality-filtered dataset contained 6,879,264 sequences, with a mean of 19,882 sequences per sample (standard deviation [SD]:14,362). After rarefying to 3000 reads per sample, 340 of the original 346 faecal samples were retained, containing 591 unique OTUs (13 phyla, 27 families, 80 genera).

**Fig. 2:**
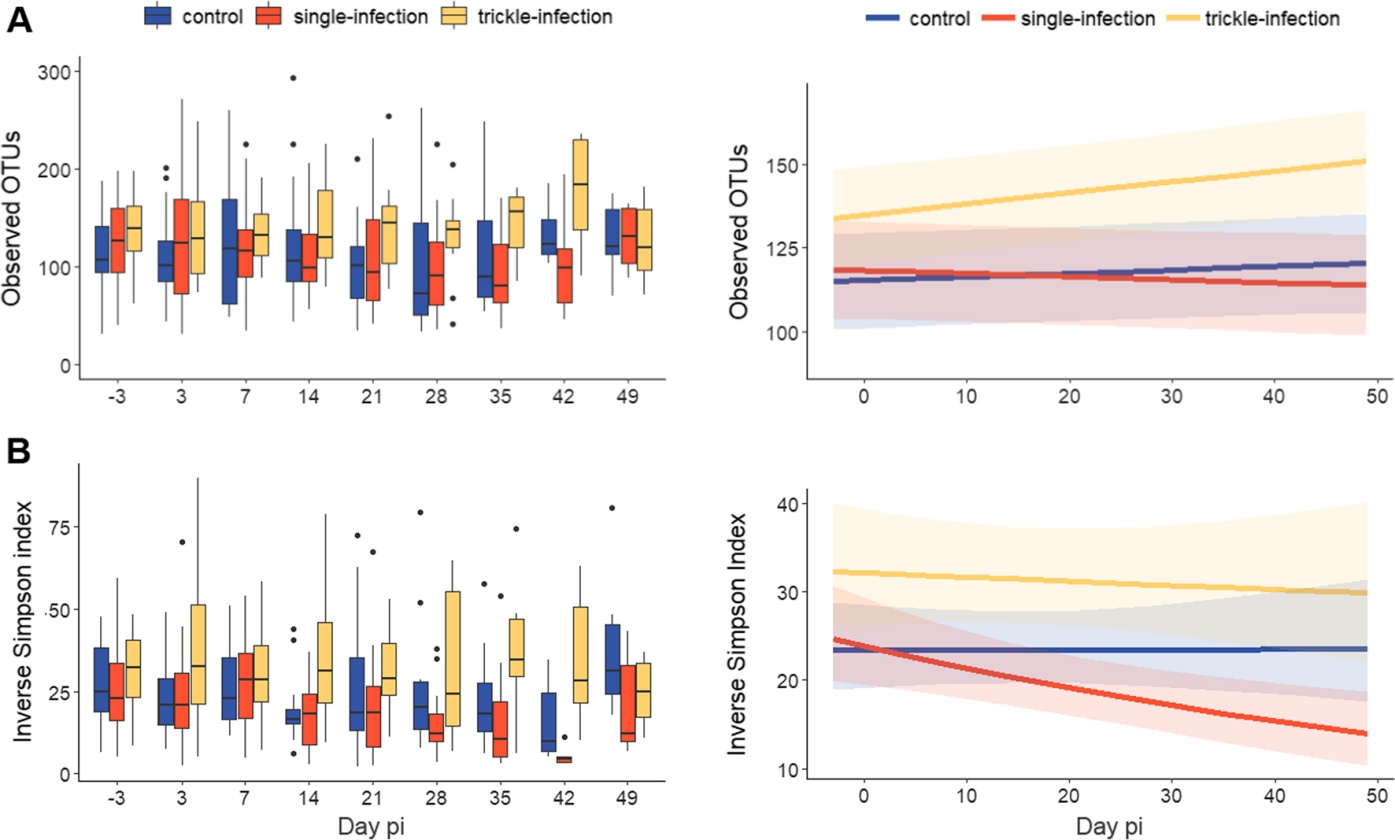
Microbial diversity (**A** observed OTUs, **B** inverse Simpson index) in faecal samples of *A. suum*-infected and non-infected control pigs. Shown are boxplots of original values (left) with boxes extending from the 25th to the 75th percentile, a line at the median, whiskers extending to 1.5 the interquartile range and dots indicating outliers; and GLMM-derived estimates with shaded areas indicating 95% confidence intervals (right). Note the different *y*-axis limits. The GLMMs indicated significantly higher inverse Simpson indices in the trickle-infection than the control group (*P* = 0.024) as well as a significant reduction in inverse Simpson indices over time in the single-infection group (*P* < 0.001)

Analysis of alpha diversity indices by GLMMs indicated a significant interaction effect of day p.i. and the *A. suum* infection group regarding the inverse Simpson index, with a significant decline in the single-infected group versus the control group over the course of the infection. In contrast, trickle-infected pigs showed a consistently higher inverse Simpson index during the entire study period (Table 1, Fig. 2). No significant differences regarding the number of observed OTUs, the Chao1 index or the Shannon index were detected (Table 1).

**Table 1:** Influence of *A. suum* infection group and day post-infection on faecal microbial alpha diversity

	Observed OTUs (Poisson error/identity link)				Chao1 index (gamma error/identity link)				Shannon index (gamma error/identity link)				Inverse Simpson index (gamma error/log link)			
	Est	SE	*z*	*P*	Est	SE	*t*	*P*	Est	SE	*t*	*p*	Est	SE	*t*	*p*
Intercept	115.36	7.51	15.36	**< 0.001**	122.32	10.05	12.17	**< 0.001**	3.77	0.11	34.03	** < 0.001**	3.15	0.10	31.95	** < 0.001**
Day p.i.	0.10	0.07	1.55	0.122	0.00	0.23	0.01	0.993	−0.01	0.16	−0.08	0.934	0.00	0.00	0.16	0.876
Group: single vs control	1.43	10.96	0.13	0.897	−0.18	14.22	−0.01	0.990	0.29	0.17	1.79	0.073	0.04	0.14	0.27	0.791
Group: trickle vs control	20.47	11.10	1.84	0.065	17.49	14.70	1.19	0.234	0.00	0.00	−0.14	0.888	0.34	0.15	2.25	**0.024**
Day p.i.*Group (single vs control)	-0.16	0.09	-1.72	0.086	−0.03	0.31	-0.09	0.929	−0.01	0.00	−1.48	0.140	−0.01	0.00	−3.81	** < 0.001**
Day p.i.*Group (trickle vs control)	0.22	0.11	1.70	0.089	0.35	0.41	0.844	0.399	0.00	0.00	−0.22	0.830	0.00	0.00	−0.84	0.400

GLMMs were carried out to analyse the influence of the *A. suum* infection group (single-infection, trickle-infection, non-infected control) and day post-infection (day p.i.) on different alpha diversity metrics. Significant *P*-values are printed in bold. Est.: estimate; OTUs: operational taxonomic units; p.i.: post-infection; SE: standard error

Microbial community composition was significantly affected by day p.i., experimental group and their interaction (PERMANOVA of JSDs, Table 2). Therefore, separate PERMANOVAs were calculated for each infection group versus the control group, and for each sampling day. Results revealed that the overall microbiota composition of the single-infected group differed significantly from the control group on days 14 and 21, whereas the trickle-infected group consistently showed a significantly different microbiota composition from that of the control group, except on day 28 p.i. (Additional file 1).

**Table 2:** PERMANOVA of porcine faecal microbiota composition

Term	*df*	SS	MS	*F*	*R*^2^	*P*
Group	2	2.39	1.19	8.00	0.04	**< 0.001**
Day p.i.	1	1.97	1.97	13.22	0.04	**< 0.001**
Group*Day p.i.	2	1.30	0.65	4.36	0.02	**< 0.001**
Residuals	334	49.76	0.15		0.90	
Total	339	55.41			1.00	

PERMANOVA was carried out to test the effect of the experimental *A. suum* group (single-infection, trickle-infection, non-infected control) and day post-infection (p.i.) on microbiota composition in porcine faecal samples, based on Jensen–Shannon distances. Significant *P*-values are printed in bold. *df* degrees of freedom; MS: mean squares; p.i.: post-infection; SS: sum of squares

#### Shifts in taxonomic composition

The dominant phyla in pig faeces were Firmicutes and Bacteroidetes, with mean relative abundance of 52.7% (SD: 15.4%) and 33.1% (SD: 12.1%) per sample, respectively. Regarding relative microbial abundance at the phylum level, only a few differences were noted between the groups. On day 3 p.i., the trickle-infected group had significantly higher relative abundance of Fibrobacteres than the control group (Kruskal–Wallis *χ*^2^ = 18.9, *df* = 2, *P*_adj_ = 0.008/Dunn’s test *P* = 0.007), while on day 14 p.i., Verrucomicrobia were significantly increased in the trickle-infected group as compared with control animals (Kruskal–Wallis *χ*^2^ = 17.8, *df* = 2, *P*_adj_ = 0.008/Dunn’s test *P* = 0.026). Significant differences at the genus level were mainly observed in the trickle-infected group, with lower *Dorea* but higher *Peptococcus* relative abundance before infection. Thereafter, differences included increased relative abundance of *Bifidobacterium*, *Fibrobacter* and an unclassified Clostridia genus on day 3 p.i., an unclassified Prevotellaceae genus on day 7 p.i., an unclassified Eubacteriaceae and unclassified Verrucomicrobia genus on day 14 p.i. and *Campylobacter* on day 21 p.i. (Table 3). Decreased relative abundance was observed for *Bifidobacterium* on days 28 and 35 p.i. In the single-infected group, *Peptococcus* was increased before infection, and an increase in *Streptococcus* was observed on days 28 and 35 p.i. No significant differences were observed on days 42 and 49 p.i. at the genus level.

**Table 3:** Genus-level differences in the faecal microbiota between *A. suum* single-infected (SI), trickle-infected (TI) and non-infected (C) pigs

	Mean relative abundance ± SD			Kruskal–Wallis test			Dunn’s test	
	SI	TI	C	*Χ*^2^	*df*	*P*_FDR-adj_	*P* (SI vs. C)	*P* (TI vs. C)
Day −3								
*Dorea* (Lachnospiraceae)	1.00 ± 0.67	0.20 ± 0.40	1.42 ± 1.41	16.65	2	0.033	0.901	**< 0.001**
*Peptococcus* (Peptococcaceae 1)	0.21 ± 0.18	0.34 ± 0.32	0.06 ± 0.12	15.37	2	0.033	**0.010**	**< 0.001**
Day 3 p.i.								
*Bifidobacterium* (Bifidobacteriaceae)	0.00 ± 0.00	1.29 ± 2.99	0.00 ± 0.00	15.10	2	0.033	1.000	**0.001**
*Fibrobacter* (Fibrobacteraceae)	0.01 ± 0.03	0.76 ± 1.31	0.21 ± 0.57	18.89	2	0.033	0.232	**0.007**
Unclassified Clostridia	0.06 ± 0.12	1.31 ± 0.25	0.04 ± 0.13	15.80	2	0.033	0.595	**< 0.001**
Day 7 p.i.								
Unclassified Prevotellaceae	0.25 ± 0.48	1.30 ± 1.02	0.31 ± 0.42	16.50	2	0.033	0.776	**0.002**
Day 14 p.i.								
Unclassified Eubacteriaceae	0.26 ± 0.43	0.98 ± 0.75	0.16 ± 0.19	15.6	2	0.033	0.999	**< 0.001**
Unclassified Verrucomicrobia	0.04 ± 0.12	1.81 ± 2.66	0.41 ± 0.80	16.20	2	0.033	0.169	**0.024**
Day 21 p.i.								
*Campylobacter* (Campylobacteraceae)	0.25 ± 0.79	1.24 ± 1.22	0.1 ± 0.24	15.6	2	0.033	0.963	**< 0.001**
Day 28 p.i.								
*Bifidobacterium* (Bifidobacteriaceae)	3.25 ± 7.49	0.00 ± 0.00	1.76 ± 2.00	14.7	2	0.035	0.503	**0.013**
*Streptococcus* (Streptococcaceae)	1.29 ± 1.54	0.06 ± 0.18	0.01 ± 0.02	16.3	2	0.033	**< 0.001**	0.995
Day 35 p.i.								
*Bifidobacterium* (Bifidobacteriaceae)	3.24 ± 4.31	0.00 ± 0.00	3.14 ± 4.04	15.8	2	0.033	0.588	**0.007**
*Streptococcus* (Streptococcaceae)	2.77 ± 4.15	0.27 ± 0.59	0.07 ± 0.22	15.0	2	0.033	**< 0.001**	0.831

Only genera that were significantly different between groups (*P*-values printed in bold) are shown. No significant differences were noted on days 42 and 49 p.i. C: control; *df*: degrees of freedom; FDR: false discovery rate; p.i.: post-infection; TI: trickle infection; SD: standard deviation; SI: single infection

**Fig. 3:**
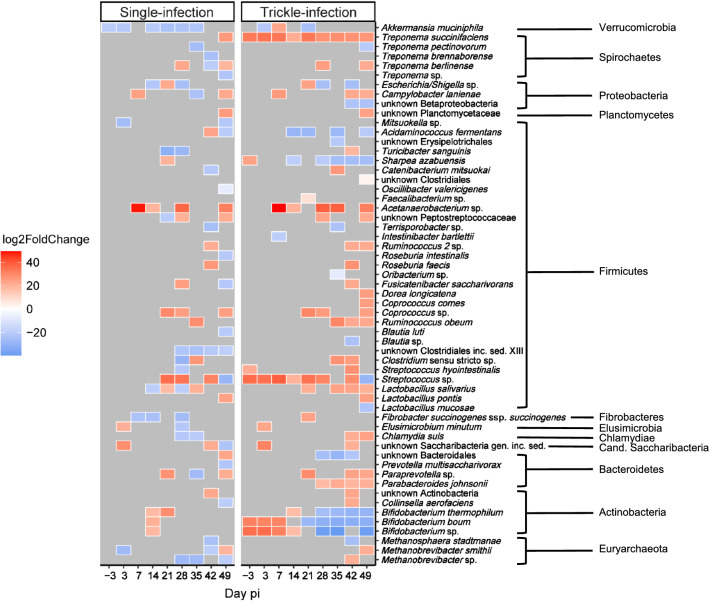
Heatmap of differentially abundant species in the faecal microbiota of *A. suum*-infected pigs compared with a non-infected control group, as determined by DESeq2 analysis. Only differences with Benjamini–Hochberg adjusted *P*-values ≤ 0.01 are shown as coloured tiles

DESeq2 analysis indicated that in faecal samples of both infection groups, the number of differentially abundant bacterial species, as compared with the control group, gradually increased during the course of the experiment. At the species level, between 1 (day −3) and 24 (day 49) species were significantly altered in abundance (Benjamini–Hochberg adjusted *P* ≤ 0.01) in the single-infected group as compared with the control group, while this number ranged from 6 (day −3) to 27 (day 49) in the trickle-infected group (Fig. 3). In both groups, a prominent increase in an *Acetanaerobacterium* sp. was noted on day 7 p.i. A further similarity was an increase in a *Coprococcus* sp. on days 21, 28 and 49 p.i. Notably, in the trickle-infected group, several species assigned to the genus *Bifidobacterium* decreased in abundance as from day 21 p.i., while members of the genera *Dorea*, *Coprococcus*, *Ruminococcus*, *Lactobacillus, Paraprevotella* and *Parabacteroides* showed an increase towards the end of the observation period (Fig. 3).

#### Faecal microbial association networks

**Fig. 4:**
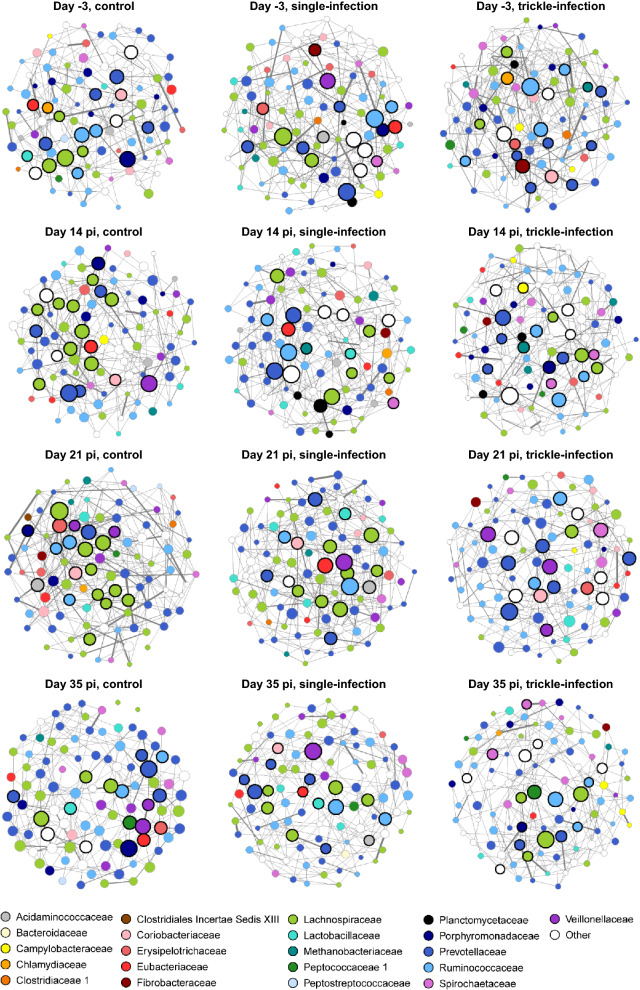
Association networks of the faecal microbiota of *A. suum* single-infected and trickle-infected pigs on day −3 (prior to infection) and on days 14, 21 and 35 p.i., compared with a non-infected control group. Only the top 100 nodes (i.e. OTUs) with the largest eigenvector centrality and only positive associations are shown. Node colour indicates taxonomy at the family level, while node size is proportional to eigenvector centrality. Hub nodes (based on eigenvector centrality) are highlighted by a bold rim

The filtered dataset used for network analyses contained 375 OTUs, which occurred in at least 20% of faecal samples. Due to the exclusion of some animals, networks were based on 15 to 18 (days −3, 14, 21) or 10 to 12 faecal samples (day 35). Network characteristics are summarized in Table 4, and partial networks (100 most central nodes only) are visualized in Fig. 4. Network density was similar in all three experimental groups from day −3 through day 21 p.i., but decreased in the control and single-infected group on day 35 p.i., while network modularity, clustering coefficient and the proportion of positive associations showed the opposite pattern. In the trickle-infected group, only the clustering coefficient and modularity showed an increase on day 35 p.i., while the remaining measures were comparable to the preceding time points. Thus, networks from the control and single-infected groups on day 35 p.i. showed a higher degree of partitioning into groups of well-connected (i.e. associated) OTUs than the trickle-infected group.

**Table 4:** Characteristics of microbial association networks determined from faecal samples of *A. suum*-infected and non-infected pigs

	Day −3			Day 14 p.i.			Day 21 p.i.			Day 35 p.i.		
	C	SI	TI	C	SI	TI	C	SI	TI	C	SI	TI
Number of nodes	363	359	370	369	350	366	365	359	364	346	324	350
Edge density	0.022	0.026	0.027	0.028	0.023	0.023	0.025	0.021	0.024	0.017	0.016	0.021
Positive edges (%)	82.48	76.193	75.73	76.326	80.675	77.228	80.427	82.619	77.997	86.944	89.260	76.471
Clustering coefficient	0.089	0.123	0.119	0.093	0.121	0.104	0.113	0.118	0.109	0.184	0.230	0.184
Modularity	0.38	0.355	0.340	0.326	0.394	0.378	0.368	0.411	0.364	0.527	0.568	0.442
No. of modules (greedy algorithm)	10	11	8	8	11	14	10	11	9	14	16	15
Mean normalized eigenvector centrality (± SD)	0.404 (± 0.213)	0.358 (± 0.219)	0.348 (± 0.227)	0.468 (± 0.216)	0.305 (± 0.215)	0.352 (± 0.219)	0.318 (± 0.210)	0.276 (± 0.220)	0.412 (± 0.227)	0.214 (± 0.196)	0.209 (± 0.210)	0.273 (± 0.198)

C: control; SI: single infection; p.i.: post-infection; SD: standard deviation; TI: trickle infection

In each network, between 17 and 19 OTUs were identified as “hub nodes” based on eigenvector centrality, i.e. OTUs with a high degree of association to other OTUs, which were themselves highly connected. These OTUs can be regarded as influential in the network, affecting the overall OTU composition. Prior to infection (day −3), the set of hub nodes was rather similar among the three experimental groups, with two hub OTUs shared by the single-infected and the control group, four by the trickle-infected and the control group and two between the single- and trickle-infected groups (Additional file 2). Between-group similarity decreased on day 14 p.i., with only two hub nodes each shared between the single-infected and the trickle-infected and the control group, respectively. On day 21 p.i., the single-infected group shared six hub OTUs with the control group, while the trickle-infected group shared only one hub OTU (assigned to *Olsenella scatoligenes*) with both other groups. On day 35 p.i., both infection groups shared one hub OTU each with the control group.

The temporal stability of hub nodes in the control group was low at the beginning of the study, with only one common hub node identified in the networks from days 14 and 21 p.i., but four hub nodes, mainly from the family Lachnospiraceae, remained consistent from day 21 to day 35 p.i. (Additional file 2). In the infection groups, temporal stability was rather low throughout the course of infection. In the single-infected group, one hub node each remained consistent from days 14 to 21 p.i. and days 21 to 35 p.i. In the trickle-infected group, two hub nodes remained consistent from day −3 to day 14 p.i. and one from days 14 to 21 p.i., but none from days 21 to 35 p.i.

In terms of hub OTU taxonomy, the most well-represented families in the control group and single-infected group were Lachnospiraceae and/or Prevotellaceae, except for day −3 and day 14 p.i., when the set of hub nodes in the single-infected group was taxonomically rather diverse (Fig. 4, Additional file 2). Most hub nodes of the trickle-infected group were assigned to Prevotellaceae and Ruminococcaceae prior to infection, while on day 14 p.i., Prevotellaceae, Lachnospiraceae and Ruminococcaceae were almost equally represented. Prevotellaceae and Veillonellaceae predominated on day 21 p.i., followed by Lachnospiraceae and Ruminococcaceae on day 35 p.i. Therefore, the trickle-infected group displayed the lowest temporal stability in terms of hub node taxonomy at the family level.

### Intestinal content

#### Alpha and beta diversity

Several ingesta samples, especially from the jejunum, had to be excluded due to insufficient DNA for sequencing or low sequencing output. Consequently, 106 of 138 small intestine samples and 96 of 98 large intestine samples were retained after quality filtering, yielding a total of 3,419,792 (mean 32,262 ± 15,440 per sample) and 2,852,943 (mean 29,718 ± 14,289) sequences, respectively. Rarefaction to 8000 reads/sample resulted in the removal of four further jejunum samples. In the small intestine, the rarefied sequences were assigned to 255 unique OTUs (8 phyla, 44 families, 84 genera), whereas 469 unique OTUs (9 phyla, 32 families, 71 genera) were present in the large intestine samples.

**Fig. 5:**
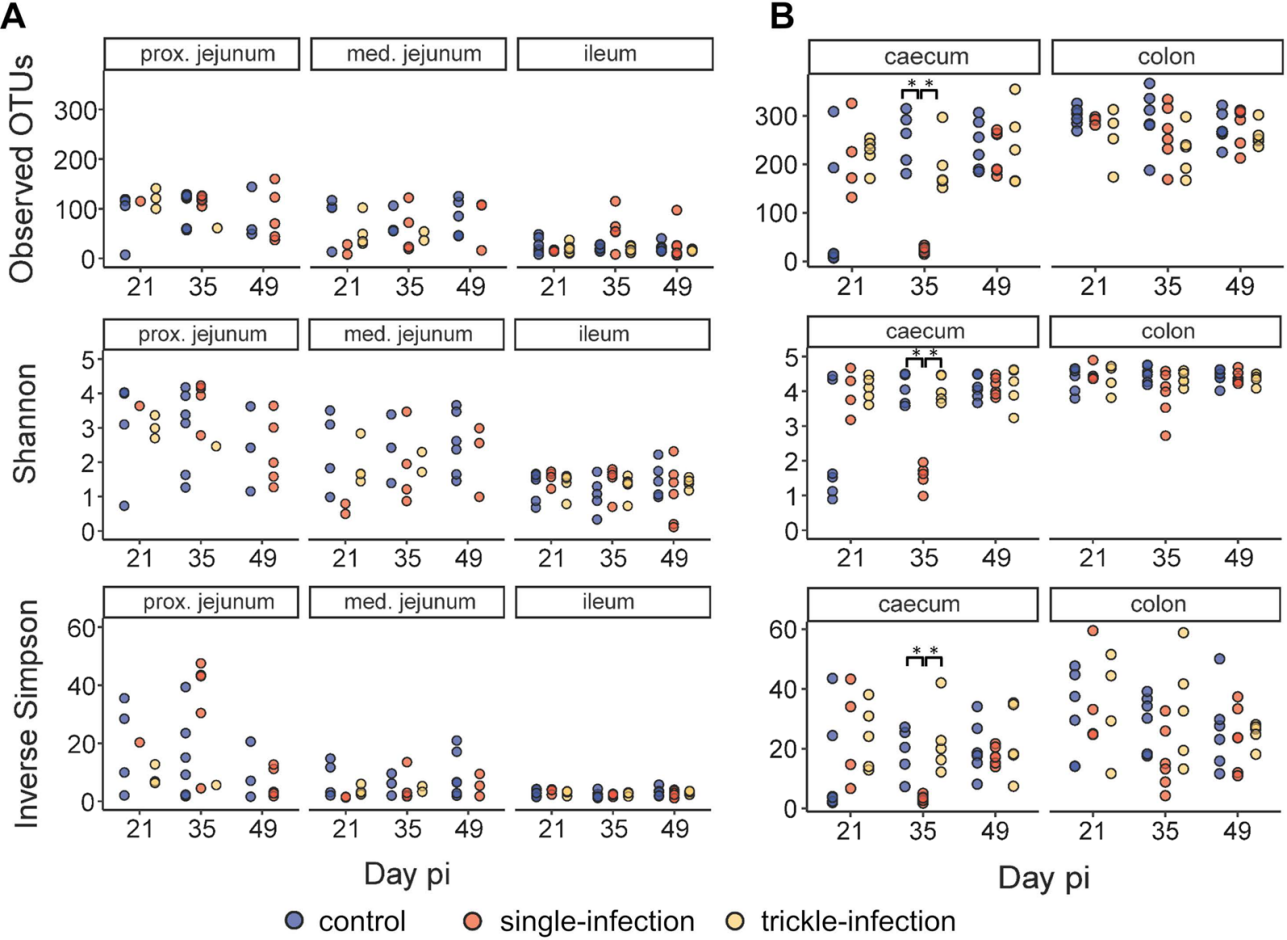
Microbial diversity (observed OTUs, Shannon index and inverse Simpson index) in the small intestine (**A**) and large intestine (**B**) of *A. suum*-infected and non-infected control pigs. Asterisks indicate statistically significant differences (Kruskal–Wallis test, FDR-corrected *P* < 0.05)

A wide range in alpha diversity among ingesta samples from the jejunum and a general decrease in diversity from the proximal to the distal small intestine were noted (Fig. 5). However, comparability between groups was limited due to the low number of successfully sequenced jejunum samples, and no significant differences in alpha diversity measures were detected in the small intestine (Kruskal–Wallis tests, FDR-adjusted *P* > 0.05). Regarding the large intestine, a significant reduction in observed OTUs, Shannon index and inverse Simpson index were noted on day 35 p.i. in the caecum of the single-infected group relative to the other groups (Kruskal–Wallis *χ*^2^ = 11.58/10.59/10.59, *df* = 2, FDR-adjusted *P* = 0.003/0.005/0.005, Dunn’s test *P* = 0.002/0.012/0.012). In fact, only 24.0 OTUs (SD: 6.4) were detected on average in the caecum samples of the single-infected group on day 35, as opposed to 252.2 (SD: 56.1) in the control and 196.2 (SD: 58.8) in the trickle-infected group. No significant differences in alpha diversity were detected regarding the colon.

**Fig. 6:**
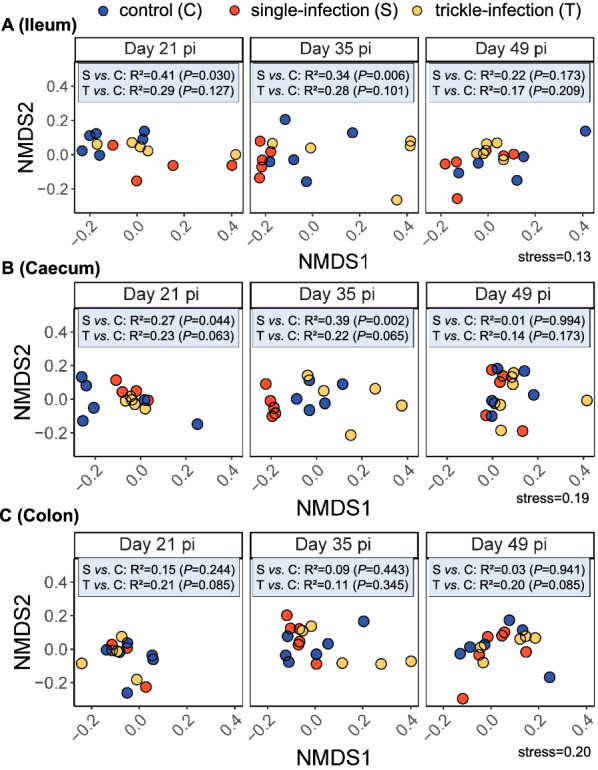
Non-metric multidimensional scaling (NMDS) plot of microbial community composition in the ileum (**A**), caecum (**B**) and colon (**C**) of *A. suum*-infected and non-infected pigs. Results of PERMANOVAs are shown in blue boxes. *S* single-infection group, *T* trickle-infection group, *C* non-infected control group

Because of the low number of jejunum ingesta samples, beta diversity analyses were not carried out for this sample subset. Regarding the other intestinal compartments, initial PERMANOVA indicated a significant main effect of group for the ileum and caecum, as well as a significant effect of day p.i. for the caecum and colon and a significant interaction effect of group and day p.i. in the ileum (Table 5). Conducting PERMANOVAs for each of the infection groups versus the control group and for each day p.i. separately revealed significant differences in microbiota composition between the single-infected group and the control group on days 21 and 35 p.i. in the ileum and caecum, whereas no significant differences were found on day 49 p.i. Regarding the trickle-infected group, no significant differences in overall microbiota composition relative to the control group were found in any intestinal compartment (Fig. 6; Additional file 3).

**Table 5:** PERMANOVAs of microbiota composition in ingesta samples from the ileum, caecum and colon

Compartment	Term	*df*	SS	MS	*F*-value	*R*^2^	*P*-value
Ileum							
	Group	2	0.27	0.13	3.31	0.12	**0.037**
	Day p.i.	1	0.06	0.07	1.64	0.03	0.205
	Group*Day p.i.	2	0.24	0.12	2.99	0.11	**0.046**
	Residuals	40	1.60	0.04	-	0.74	–
	Total	45	2.17	-	-	1.00	–
Caecum							
	Group	2	0.30	0.15	2.01	0.08	**0.028**
	Day p.i.	1	0.37	0.37	4.97	0.09	** < 0.001**
	Group*Day p.i.	2	0.13	0.07	0.89	0.03	0.547
	Residuals	42	3.10	0.07	–	0.80	-
	Total	47	3.90	-	–	1.00	-
Colon							
	Group	2	0.17	0.08	1.67	0.07	0.078
	Day p.i.	1	0.19	0.19	3.84	0.08	**0.001**
	Group*Day p.i.	2	0.06	0.03	0.61	0.03	0.846
	Residuals	42	2.04	0.05	–	0.84	–
	Total	47	2.45	-	–	1.00	–

PERMANOVAs testing the effect of experimental group and day p.i. on the microbial composition were based on OTU-level Jensen–Shannon distance values. Significant *P*-values are printed in bold. *df*: degrees of freedom; MS: mean squares; p.i.: post-infection; SS: sum of squares

#### Shifts in taxonomic composition

In the small intestine, Firmicutes was the dominant phylum, with mean relative abundance of 90.56% per sample (SD: 11.7%). In the large intestine, Firmicutes showed a mean relative abundance of 74.6% (SD: 12.3%), while on average 19.8% (SD: 10.9%) of the sequences were assigned to Bacteroidetes.

**Fig. 7:**
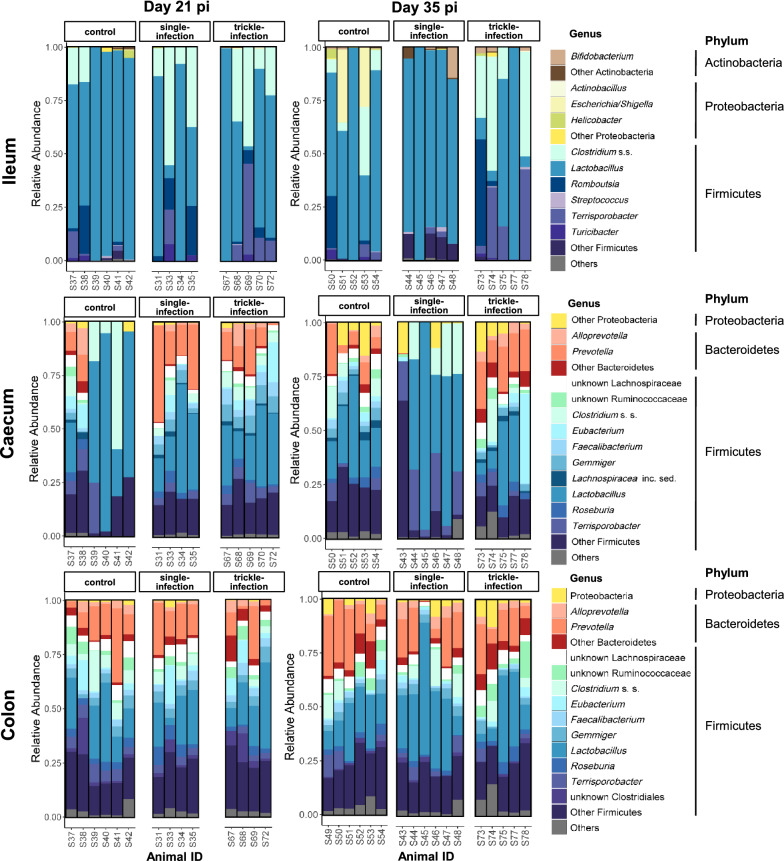
Genus-level taxonomic composition of the microbiota in the ileum, caecum and colon of *A. suum*-infected vs non-infected control pigs on days 21 and 35 p.i.

The 12 most abundant bacterial genera in the ileum, caecum and colon of pigs slaughtered at days 21 and 35 p.i. are shown in Fig. 7. The microbiota of the ileum was dominated by the genera *Lactobacillus*, *Clostridium* sensu stricto (s.s.) and *Terrisporobacter*, while the caecum and colon were dominated by *Lactobacillus*, *Prevotella* and *Clostridium* s.s., among others. At the phylum level, no statistically significant differences in relative abundance were detected among the ingesta samples of the different experimental groups. At the genus level, however, significant differences were apparent in caecum samples from days 21 and 35 p.i., but not day 49 p.i. On day 21 p.i., seven and eight genera showed a higher relative abundance in the single- and the trickle-infected groups, respectively, than in the control group, with an increase in *Megasphaera*, *Blautia*, *Fusicatenibacter* and *Holdemanella* in both infection groups (Table 6). Furthermore, *Romboutsia* was decreased in the single-infected group as compared with the control group. On day 35 p.i., 26 bacterial genera were significantly reduced in the caecum of the single-infected group (Table 6), in line with the significantly reduced alpha diversity. Four of these genera (*Fusicatenibacter*, *Ruminococcus* 2, *Collinsella* and *Holdemanella*) also showed significantly lower relative abundance in the trickle-infected group at this time point.

**Table 6:** Genus-level differences in the caecal microbiota between *A. suum* single-infected (SI), trickle-infected (TI) and non-infected (C) pigs

	Mean relative abundance (%) ± SD			Kruskal–Wallis test			Dunn’s test	
	SI	TI	C	*Χ*^2^	*df*	*P*_FDR-adj_	*P* (SI vs C)	*P* (TI vs C)
Day 21 p.i.								
*Gemmiger* (Ruminococcaceae)	4.65 ± 1.69	7.53 ± 1.3	0.57 ± 1.25	11.24	2	**0.038**	0.138	**0.002**
*Prevotella* (Prevotellaceae)	26.19 ± 14.44	10.66 ± 7.28	3.52 ± 5.58	9.24	2	**0.049**	**0.005**	0.229
*Megasphaera* (Veillonellaceae)	2.93 ± 2.22	2.24 ± 1.37	0.11 ± 0.28	10.52	2	**0.038**	**0.012**	**0.012**
*Faecalibacterium* (Ruminococcaceae)	3.02 ± 0.84	6.02 ± 1.68	0.57 ± 0.89	11.78	2	**0.038**	0.101	**0.001**
*Romboutsia* (Peptostreptococcaceae)	0.17 ± 0.11	0.41 ± 0.14	7.37 ± 10.76	11.13	2	**0.038**	**0.002**	0.132
*Blautia* (Lachnospiraceae)	2.39 ± 0.9	2.58 ± 0.78	0.3 ± 0.56	9.57	2	**0.047**	**0.026**	**0.012**
*Fusicatenibacter* (Lachnospiraceae)	0.27 ± 0.12	0.27 ± 0.14	0.03 ± 0.07	9.41	2	**0.048**	**0.017**	**0.020**
*Collinsella* (Coriobacteriaceae)	0.54 ± 0.38	0.2 ± 0.15	0.03 ± 0.06	10.37	2	**0.038**	**0.003**	0.129
*Holdemanella* (Erysipelotrichaceae)	0.22 ± 0.08	0.35 ± 0.23	0.02 ± 0.05	10.33	2	**0.038**	**0.035**	**0.006**
*Olsenella* (Coriobacteriaceae)	0.14 ± 0.08	0.23 ± 0.13	0.02 ± 0.04	9.40	2	**0.048**	0.069	**0.007**
*Peptococcus* (Peptococcaceae 1)	0.08 ± 0.11	0.07 ± 0.02	0.00 ± 0.00	9.38	2	**0.048**	0.074	**0.007**
*Acidaminococcus* (Acidaminococcaceae)	0.22 ± 0.31	0.00 ± 0.00	0 ± 0.01	11.89	2	**0.038**	**0.007**	0.893
Day 35 p.i.								
*Gemmiger* (Ruminococcaceae)	0.00 ± 0.00	2.60 ± 1.1	4.13 ± 1.57	11.95	2	**0.038**	**0.002**	0.575
*Prevotella* (Prevotellaceae)	0.06 ± 0.16	18.06 ± 4.57	9.69 ± 7.82	11.93	2	**0.038**	**0.041**	0.491
*Megasphaera* (Veillonellaceae)	0.00 ± 0.00	0.32 ± 0.58	3.90 ± 3.73	10.50	2	**0.038**	**0.003**	0.074
*Roseburia* (Lachnospiraceae)	0.01 ± 0.02	3.59 ± 1.51	3.02 ± 2.05	10.95	2	**0.038**	**0.013**	0.970
Unknown Lachnospiraceae	0.00 ± 0.00	4.25 ± 2.26	5.41 ± 1.29	11.28	2	**0.038**	**0.005**	0.918
*Butyricicoccus* (Clostridiaceae)	0.00 ± 0.00	0.30 ± 0.36	2.77 ± 1.26	12.57	2	**0.038**	**< 0.001**	0.051
*Faecalibacterium* (Ruminococcaceae)	0.00 ± 0.00	0.63 ± 0.75	3.21 ± 1.61	12.60	2	**0.038**	**< 0.001**	0.127
*Lachnospiracea* inc. sed. (Lachnospiraceae)	0.01 ± 0.02	1.80 ± 0.61	2.07 ± 1.53	10.95	2	**0.038**	**0.013**	0.970
*Phascolarctobacterium* (Acidaminococcaceae)	0.00 ± 0.00	1.36 ± 0.92	0.94 ± 0.78	11.28	2	**0.038**	**0.014**	0.918
*Blautia* (Lachnospiraceae)	0.00 ± 0.00	1.01 ± 0.62	1.76 ± 0.44	11.71	2	**0.038**	**0.001**	0.217
*Coprococcus* (Lachnospiraceae)	0.00 ± 0.00	1.03 ± 0.63	1.88 ± 1.22	10.58	2	**0.038**	**0.003**	0.430
*Dorea* (Lachnospiraceae)	0.00 ± 0.00	0.13 ± 0.1	0.56 ± 0.07	13.16	2	**0.038**	**< 0.001**	0.093
Unknown Erysipelotrichales	0.00 ± 0.00	1.16 ± 1.46	0.6 ± 0.51	13.14	2	**0.038**	**< 0.001**	0.094
Unknown Bacteroidia	0.00 ± 0.00	1.16 ± 1.46	0.60 ± 0.51	9.35	2	**0.048**	**0.017**	1.000
Unknown Clostridiales	0.03 ± 0.06	1.70 ± 2.1	1.68 ± 0.92	9.72	2	**0.046**	**0.007**	0.769
*Oribacterium* (Lachnospiraceae)	0.00 ± 0.00	0.14 ± 0.19	0.5 ± 0.34	9.92	2	**0.043**	**0.003**	0.102
*Fusicatenibacter* (Lachnospiraceae)	0.00 ± 0.00	0.05 ± 0.11	0.26 ± 0.22	9.95	2	**0.043**	**0.004**	**0.049**
*Ruminococcus* 2 (Ruminococcaceae)	0.00 ± 0.00	0.04 ± 0.08	0.38 ± 0.25	12.19	2	**0.038**	**0.002**	**0.014**
*Collinsella* (Coriobacteriaceae)	0.00 ± 0.00	0.01 ± 0.02	0.29 ± 0.16	13.03	2	**0.038**	**0.001**	**0.009**
Unknown Prevotellaceae	0.00 ± 0.00	0.69 ± 0.5	0.37 ± 0.24	11.48	2	**0.038**	**0.019**	0.805
*Holdemanella* (Erysipelotrichaceae)	0.00 ± 0.00	0.00 ± 0.00	0.28 ± 0.15	14.41	2	**0.038**	**0.001**	**0.002**
Unknown Firmicutes	0.00 ± 0.00	0.08 ± 0.07	0.26 ± 0.22	10.58	2	**0.038**	**0.003**	0.430
*Selenomonas* (Selenomonadaceae)	0.00 ± 0.00	0.41 ± 0.55	0.23 ± 0.18	11.30	2	**0.038**	**0.014**	0.918
*Anaerovibrio* (Veillonellaceae)	0.00 ± 0.00	0.48 ± 0.28	0.22 ± 0.29	11.95	2	**0.038**	**0.032**	0.575
*Paraeggerthella* (Coriobacteriaceae)	0.00 ± 0.00	0.03 ± 0.04	0.11 ± 0.05	10.82	2	**0.038**	**0.002**	0.062
Unknown Coriobacteriaceae	0.00 ± 0.00	0.13 ± 0.13	0.28 ± 0.18	11.10	2	**0.038**	**0.002**	0.312

Only genera that were significantly different between groups (*P*-values printed in bold) are listed. No significant differences were noted on day 49 p.i. C: control; *df*: degrees of freedom; FDR, false discovery rate; p.i., post-infection; TI: trickle infection; SD: standard deviation; SI: single infection

At the species level, DeSeq2 analysis indicated significant changes in bacterial abundance in the ileum, caecum and colon of both infection groups (Additional file 4). The prevailing pattern across all three intestinal compartments was that of a lower abundance of many taxa relative to the control group, except for the ileum of the single-infected group on days 35 and 49 p.i. Several species in ileal ingesta showed increased abundance on day 35 p.i. in this group, including *Bifidobacterium thermophilum*, *Eubacterium coprostanoligenes*, *Lactococcus raffinolactis* and unknown species of *Oscillibacter*, *Veillonella*, *Streptococcus*, Ruminococcaceae, Porphyromonadaceae, Demequinaceae and *Lachnospiracea* incertae sedis. *Bifidobacterium thermophilum* and the unknown *Streptococcus* sp. were also elevated in the trickle-infected group on day 35 p.i. In the single-infected group, *Eubacterium coprostanoligenes* and the unknown species of *Veillonella*, *Oscillibacter*, Ruminococcaceae and Porphyromonadaceae remained elevated on day 49 p.i.

In the caecum of the single-infected group, only a few bacterial taxa were significantly altered in abundance on days 21 and 49 p.i., while 74 bacterial taxa were decreased in abundance and only two (*Uruburuella suis* and an unknown *Streptococcus* sp.) were increased on day 35 p.i. *Uruburuella suis* remained significantly increased in this group on day 49 p.i., while *Lactobacillus equicursoris* was the only taxon to remain decreased from day 35 to day 49 p.i. (Additional file 4).

In contrast, only minor changes were observed at the species level in the caecum of the trickle-infected group and in the colon of both infection groups. However, considering all studied intestinal compartments (ileum, caecum, colon) and all time points, the following parallels seem noteworthy: On day 35 p.i., an unknown *Streptococcus* sp. was elevated in all three compartments of both infection groups, except in the caecum of the trickle-infected group. In addition, a decrease in *L. equicursoris* was observed on day 49 p.i. in all compartments of both infection groups. The decrease in various *Bifidobacterium* spp. on day 49 p.i. was a common pattern in all compartments of the trickle-infected group, as well as in faecal samples (see section above).

## Discussion

The present study examined faecal and ingesta samples from different intestinal segments to characterize changes in the intestinal microbiota of pigs during the first 7 weeks of *A. suum* infection. Unfortunately, the sequencing success of samples from the jejunum, where *A. suum* resides, was low, limiting the possibility of making meaningful comparisons between experimental groups for this intestinal compartment. Bacterial abundance is rather low in the small intestine [38], and low sequencing success has also been a problem in previous studies [e.g. 39, 40].

### Alpha diversity was differentially affected by *A. suum* single and trickle infection

The current study used young pigs of a few weeks to approximately 3 months of age (to mimic infections in fattening farms) whose intestinal microbiota is still developing. Previous studies have reported both an increase [41] and a decrease [42] in microbial alpha diversity in healthy pigs during this time span. Here, a marginal increase in observed OTUs and inverse Simpson index was observed over time in the non-infected control group. In contrast, a significant decrease in alpha diversity, more precisely in the inverse Simpson index, which takes microbial abundance into account, occurred in faecal samples of pigs single-infected with 10,000 *A. suum* eggs. Further, a dramatic decrease in all examined alpha diversity measures was observed in the caecum of the single-infected group at day 35 p.i. A decrease in microbial diversity was also observed in the colon contents of pigs 54 days after infection with 300 *A. suum* eggs each on three consecutive days, independent of the intestinal worm burden [19]. In the current study, however, colonic microbial diversity was not significantly affected. The apparent discrepancy between alpha diversity analyses of colon and faecal samples may be due to the smaller sample sizes regarding ingesta, reducing statistical power. Furthermore, alpha diversity in the caecum was restored by day 49 p.i. *Ascaris suum* excretory–secretory (ES) products include a suite of antimicrobial factors, possibly to actively manipulate its microbial environment. Interestingly, the spectrum of antimicrobial products changes throughout the parasite`s life cycle, with a higher diversity of antimicrobial components detected in adult parasites compared with L4 [15]. Moulting of L4 occurs around days 21–29 p.i.; thus, the sharp drop in alpha diversity observed at day 35 p.i. in the caecum of single-infected pigs may be due to the recent development of L4 to the pre-adult stage. However, it remains speculative why this sharp reduction in alpha diversity was not observed in the other intestinal compartments. Most of the significantly reduced genera in the caecum belonged to the families Lachnospiraceae and Ruminococcaceae, which are generally more abundant in the porcine large intestine than in the small intestine [43, 44]. It is conceivable that the *A. suum* ES antigen preferentially affects these taxa, which would explain why no alpha diversity reduction was seen in the ileum. Furthermore, the amount of antimicrobial products reaching the colon may be too small or degenerated to cause comparable effects. In addition, it remains an open question which mechanisms lead to diversity restoration between days 35 and 49 p.i. The so-called self-cure reaction, which causes partial elimination of the worm burden, takes place earlier, approximately between days 17 and 21 following a single experimental infection [5], and is thus unlikely to be responsible for the restoration of alpha diversity in the caecum.

In contrast to the single-infected group, no significant alpha diversity changes were noted in the different intestinal compartments of the trickle-infected group, while alpha diversity in faecal samples was higher than in the control group throughout the course of the study, including before infection. Previous studies have reported mixed results regarding microbial alpha diversity during helminth infections. Consequently, a recent meta-analysis identified no significant differences between helminth-infected and non-infected hosts, apart from rodents, which showed decreased microbial alpha diversity upon helminth infection [45]. In contrast, increased alpha diversity has been reported in humans naturally infected with soil-transmitted helminths, including *Ascaris* spp., in different parts of the world [e.g. 46–48]. These ambiguous results may be driven by differential effects of experimental, high-dose infections versus continuous trickle infections, aside from species-specific differences between host–parasite systems. In consequence, care should be taken when extrapolating results from experimental infection studies using a single infection dose to natural scenarios.

### *Ascaris suum* predominantly affected the microbiota composition of the caecum

The microbiota composition of the caecum was predominantly affected by *A. suum* infection, as indicated by significant differences in the relative abundance of various microbial genera. On day 21 p.i., an increase in several short-chain fatty acid (SCFA)-producing genera was noted in both infection groups in this intestinal compartment. The opposite was observed on day 35 p.i., with a significantly reduced abundance of several SCFA-producing genera in both infection groups. Specifically, *Blautia*, *Fusicatenibacter*, *Holdemanella* and *Megasphaera* were significantly increased in both infection groups on day 21 p.i., while the trickle-infected group additionally displayed an increase in *Faecalibacterium*. *Fusicatenibacter*, *Ruminococcus* 2, *Collinsella* and *Holdemanella* were reduced in both groups on day 35 p.i. Increased production of SCFAs such as butyrate and propionate has been observed in a variety of intestinal helminth infections and is regarded as beneficial for both the parasites and the host, attenuating inflammation and serving as an energy source for intestinal epithelial cells [2, 49, 50]. The shift from an increase to a decrease in SCFA-producing taxa may again be related to the moulting of L4 to adult parasites around days 21–29 p.i., and the accompanying shift in the spectrum of antimicrobial products [15]. SCFA production may be especially beneficial for the parasite during its L4 stage, when it is vulnerable to the “self-cure reaction”, which is characterized by an increase in mucosal eosinophils, macrophages, T-cells and, consequently, smooth muscle contractility [6]. SCFAs are known to modulate T-cell function [51], and may attenuate the self-cure reaction at a critical time point for the parasite, while older parasites are able to counteract the increased peristaltic movements due to their large size and strong musculature [6]. In addition, it has been suggested that adult *A. suum* themselves may induce a strong downregulation of inflammatory pathways in the porcine intestinal mucosa during later stages of infection [52].

In line with the reduction in alpha diversity on day 35 p.i. in the caecum of the single-infected group discussed above, various other taxa were affected. Overall, 26 bacterial genera showed a significantly reduced abundance, including for example *Prevotella, Roseburia* and *Gemmiger*, in addition to the above-mentioned genera. *Prevotella* is considered a keystone taxon in the large intestine of pigs, with a profound influence on the community structure and functioning of the microbiota [53]. Indeed, OTUs assigned to *Prevotella* were frequently identified as “hub nodes” in the faecal microbiota networks of the present study. In the caecum on day 35 p.i., the relative abundance of this genus amounted to 9.7% and 18.1% on average in the control group and the trickle-infected group, respectively, but only 0.1% in the single-infected group. The genus *Prevotella* has been positively associated with production parameters such as feed intake, feed conversion efficiency and weight gain, possibly due to their ability to ferment complex carbohydrates [54]. Thus, an *A. suum*-related depletion of *Prevotella* in the caecum might contribute to the lower feed intake and weight gain observed in some studies on *A. suum*-infected pigs [e.g. 55, 56]. However, in a previous study on *A. suum*-infected pigs, Wang et al. [19] rather observed an increase in *Prevotella* in colon contents at day 54 p.i., while the significant genus-level reduction in *Prevotella* in the current study occurred only transiently in the caecum, and not in the colon or the faeces, and was limited to the single-infected group. In terms of weight gain, we did not observe significant differences between the single-infected and the control group at any time point, whereas higher weight gain was indeed noted in the trickle-infected pigs, in line with the rather high *Prevotella* abundance in this group on day 35 p.i. However, the difference in weight gain was statistically significant only on day 49 p.i., when no significant differences in *Prevotella* abundance were evident, possibly because of the transient nature of the microbial alterations.

### *Ascaris suum* infection modulated bacterial taxa with probiotic properties

As a result of the significant reduction in various taxa, the caecal microbiota of single-infected pigs was mainly characterized by members of the genus *Lactobacillus* on day 35 p.i., although the increase in relative abundance of *Lactobacillus* was not statistically significant. Since next-generation sequencing data only allow the assessment of relative and not absolute abundance, it remains unclear whether *Lactobacillus* populations had indeed expanded, or whether they remained constant and overall absolute microbial abundance was reduced. Interestingly, Andersen-Civil et al. [18] observed a similar expansion of the genus *Lactobacillus* in the jejunum and colon of *A. suum*-infected pigs at day 14 p.i. Since a decrease in lactase activity has been observed during *A. suum* infection [57], and lactose intolerance has also been reported in *A. lumbricoides*-infected children [58], higher lactose levels in the intestinal contents of infected pigs might boost lactic acid bacteria such as *Lactobacillus*. Alternatively, it has been suggested that increased mucus secretion during helminth infection may favour lactobacilli [18]. An increase in *Lactobacillus* has also been repeatedly reported in the gut microbiota of helminth-infected rodents [45] and in *T. suis*-infected pigs [59]. Further, it has been suggested that *Lactobacillus* spp. might confer a survival advantage to *Heligmosomoides polygyrus* in mice [60] and *Trichuris* spp. in pigs [59]. Like SCFAs, lactate may downregulate proinflammatory responses in the gut [61]. However, on the species level, no clear picture regarding *Lactobacillus* was observed, as an increase in *L. salivarius* and *L. pontis* occurred in faecal samples at some time points as from day 21 p.i., whereas in ingesta samples rather a decrease in certain *Lactobacillus* spp. was observed. Specifically, *L. equicursoris* was decreased in the ileum, caecum and colon on day 35 p.i. (single-infected group only) and day 49 p.i. (both infection groups), and *L. mucosae* in the ileum on days 35 and 49 p.i. (trickle-infected group only).

One striking alteration observed in the trickle-infected group was the reduction in several *Bifidobacterium* spp., including *B. boum* and *B. thermophilum*, in faecal samples (day 28 p.i. onwards) and in ingesta samples from the ileum, caecum and colon (day 49 p.i.). In a recent meta-analysis, a reduction in the genus *Bifidobacterium* was identified as a common feature across different helminth–host systems [45]. Like *Lactobacillus* spp., members of the genus *Bifidobacterium* are considered probiotic, beneficial bacteria [62]. Recently, *Bifidobacterium animalis* ssp. *lactis* was shown to exert a protective effect during *A. suum* infection, attenuating the parasite’s effect on glucose transport as measured in Ussing chambers and modulating the immune response towards increased anti-*A. suum* antibody production [12]. Along these lines, feeding of a fructan-rich chicory-based diet to piglets decreased *A. suum* burden, while increasing the abundance of *Bifidobacterium* spp. in the ileum [63]. Therefore, reducing *Bifidobacterium* populations might be considered a survival strategy for the parasite.

### *Ascaris suum* affected temporal stability of faecal microbial association networks

Faecal microbial association networks showed a low degree of temporal stability in the first 3 weeks of the study in all three experimental groups, in line with the dynamic nature of the gut microbiota in growing pigs [41]. However, in the control group and the single-infected group, a tendency towards stabilization of the networks was observed between days 21 and 35 p.i., indicated by a decrease in network density and a higher degree of partitioning into groups of well-connected OTUs. Furthermore, the identity of several influential OTUs (hub nodes) remained consistent in the control group from day 21 to 35. The control group and the single-infected group also exhibited a higher degree of similarity in terms of hub node identity (shared hub nodes) and family membership of hub nodes, especially on days 14 and 21 p.i. In contrast, the trickle-infected group shared fewer hub OTUs with the control group and displayed little temporal stability in terms of hub node taxonomy over time. In general, most hub nodes in the microbial networks from the current study were members of Lachnospiraceae, Prevotellaceae and Ruminococcaceae across groups and time points, in line with previous findings in young pigs [43]. However, Lachnospiraceae and Prevotellaceae clearly dominated in the control and single-infected group, especially from day 21 p.i. onwards, while in the trickle-infected group, the pattern was more variable over time, with Ruminococcaceae or Veillonellaceae sometimes dominating over either of the aforementioned families, and a larger proportion of varying other families contributing to the set of hub nodes. Therefore, trickle infection with *A. suum* seemed to prevent the establishment of stable microbial interaction networks until day 35 p.i. Due to sample size considerations, networks were not calculated for days 42 and 49 p.i. or for the different intestinal compartments. Therefore, further studies are required to assess whether stabilization occurs later during infection, or whether *A. suum* continues to perturb the porcine intestinal microbiota during its patent phase after trickle infection.

## Conclusions

The present study revealed marked differences regarding changes in the porcine intestinal microbiota induced by a single versus trickle infection with *A. suum*. The single infection caused a remarkable, but transient, decrease in microbial diversity in the caecum. Although this was not observed following trickle infection, an increase in SCFA-producing genera in the caecum on day 21 p.i., which shifted to a decrease on day 35 p.i., was a common finding in both groups, possibly related to changes in ES products following the parasite’s final moult. Moreover, trickle infection, but not single infection, prevented the stabilization of microbial interaction networks. The differences observed between the groups may be due to a more heterogeneous worm population in the trickle-infected group, as larvae continued to arrive in the small intestine over a longer time span. Furthermore, an inverse relationship between infection dose and successful establishment of worms in the intestine has been reported [64], but this view was later challenged [5]. The high degree of overdispersion observed regarding *A. suum* burden in experimental and natural infections may rather be due to an individual predisposition [65]. It was not possible to assess the effect of individual worm burden on the results in the current study, as the intestines were subjected to measurement of electrogenic nutrient transport immediately after slaughter, precluding intestinal washing to recover all parasites [13]. However, the study of Wang et al. [19] showed that the effect of *A. suum* on microbial diversity in the colon was independent of worm burden. Nevertheless, further studies should take this factor into account, especially when examining chronically infected pigs. The current study filled the gap between previous observations made on pigs infected for 14 days and 54 days with *A. suum* [17, 19]; however, studies on chronically infected pigs beyond this time point are still lacking.

## Supplementary Information

**Additional file 1.** Results of PERMANOVA for each day p.i. testing the effect of the *A. suum* experimental group (single-infection, trickle-infection, non-infected control) on microbiota composition in porcine faecal samples, based on Jensen–Shannon distances. Significant *P*-values are printed in bold.

**Additional file 2.** Hub nodes identified in faecal microbial association networks from *Ascaris suum* single-infected and trickle-infected pigs and a non-infected control group based on eigenvector centrality. Hubs shared between experimental groups on the same day are highlighted in yellow, while hubs occurring in subsequent networks of the same group are highlighted in green.

**Additional file 3.** Results of PERMANOVAs testing the effect of each *A. suum* experimental group vs the control group on the microbiota composition in ingesta samples from the ileum, caecum and colon on days 21, 35 and 49 p.i. Analyses were based on OTU-level Jensen–Shannon distance values.

**Additional file 4.** Heatmap of differentially abundant species in the (A) ileum and (B) caecum and colon of *A. suum*-infected pigs compared with a non-infected control group, as determined by DESeq2 analysis. Only differences with Benjamini–Hochberg-adjusted *P*-values ≤ 0.01 are shown as coloured tiles.

## Data Availability

The datasets generated during the current study are available in the National Center for Biotechnology Information Sequence Read Archive (SRA) under the SRA accession: PRJNA790705.

## Abbreviations

FDR: False discovery rate
GLMM: Generalized linear mixed model
JSD: Jensen–Shannon distance
OTU: Operational taxonomic unit
PERMANOVA: Permutational analysis of variance
p.i.: Post-infection
SCFA: Short-chain fatty acid
SD: Standard deviation
SE: Standard error
