# Supplementary material for: Modulation of the porcine intestinal microbiota in the course of Ascaris suum infection
Source: Parasit Vectors. 2022 Nov 17;15:433. doi: 10.1186/s13071-022-05535-w (PMC9673396; doi:10.1186/s13071-022-05535-w)
Supplement: Supplementary file 1 — Additional file 1. Results of PERMANOVA for each day p.i. testing the effect of the A. suum experimental group (single-infection, trickle-infection, non-infected control) on microbiota composition in porcine faecal samples, based on Jensen–Shannon distances. Significant P-values are printed in bold. [file 13071_2022_5535_MOESM1_ESM.docx]

**Additional file 1:** Results of PERMANOVA analysis for each day pi testing the effect of the *A. suum* experimental group (single-infection, trickle-infection, non-infected control) on microbiota composition in porcine faecal samples, based on Jensen-Shannon distances. Significant *P*-values are printed in bold.

|  | **Single-infection vs. control** | | | | | | **Trickle-infection vs. control** | | | | | |
| --- | --- | --- | --- | --- | --- | --- | --- | --- | --- | --- | --- | --- |
|  | **Df** | **SS** | **MS** | **F** | **R^2^** | ***P*** | **Df** | **SS** | **MS** | **F** | **R^2^** | ***P*** |
| **Day -3** |  |  |  |  |  |  |  |  |  |  |  |  |
| Group | 1 | 0.14 | 0.14 | 0.91 | 0.03 | 0.544 | 1 | 0.32 | 0.32 | 2.04 | 0.06 | **0.018** |
| Residuals | 32 | 4.90 | 0.15 |  | 0.97 |  | 31 | 4.82 | 0.16 |  | 0.94 |  |
| Total | 33 | 5.04 |  |  | 1.00 |  | 32 | 5.14 |  |  | 1.00 |  |
| **Day 3 pi** |  |  |  |  |  |  |  |  |  |  |  |  |
| Group | 1 | 0.13 | 0.13 | 0.91 | 0.03 | 0.512 | 1 | 0.46 | 0.46 | 2.99 | 0.09 | **<0.001** |
| Residuals | 32 | 4.58 | 0.14 |  | 0.97 |  | 31 | 4.81 | 0.16 |  | 0.91 |  |
| Total | 33 | 4.71 |  |  | 1.00 |  | 32 | 5.27 |  |  | 1.00 |  |
| **Day 7 pi** |  |  |  |  |  |  |  |  |  |  |  |  |
| Group | 1 | 0.19 | 0.19 | 1.33 | 0.04 | 0.173 | 1 | 0.38 | 0.38 | 2.65 | 0.08 | **<0.001** |
| Residuals | 32 | 4.52 | 0.14 |  | 0.96 |  | 30 | 4.34 | 0.14 |  | 0.92 |  |
| Total | 33 | 4.70 |  |  | 1.00 |  | 31 | 4.72 |  |  | 1.00 |  |
| **Day 14 pi** |  |  |  |  |  |  |  |  |  |  |  |  |
| Group | 1 | 0.41 | 0.41 | 2.96 | 0.09 | **0.001** | 1 | 0.72 | 0.72 | 5.20 | 0.14 | **<0.001** |
| Residuals | 31 | 4.35 | 0.14 |  | 0.91 |  | 31 | 4.27 | 0.14 |  | 0.86 |  |
| Total | 32 | 4.76 |  |  | 1.00 |  | 32 | 4.99 |  |  | 1.00 |  |
| **Day 21 pi** |  |  |  |  |  |  |  |  |  |  |  |  |
| Group | 1 | 0.41 | 0.41 | 2.54 | 0.08 | **0.005** | 1 | 0.42 | 0.42 | 2.78 | 0.08 | **0.003** |
| Residuals | 31 | 4.97 | 0.16 |  | 0.92 |  | 31 | 5.70 | 0.15 |  | 0.92 |  |
| Total | 32 | 5.37 |  |  | 1.00 |  | 32 | 5.12 |  |  | 1.00 |  |
| **Day 28 pi** |  |  |  |  |  |  |  |  |  |  |  |  |
| Group | 1 | 0.27 | 0.28 | 1.77 | 0.08 | 0.052 | 1 | 0.25 | 0.25 | 1.49 | 0.07 | 0.105 |
| Residuals | 21 | 3.28 | 0.16 |  | 0.92 |  | 19 | 3.12 | 0.16 |  | 0.93 |  |
| Total | 22 | 3.56 |  |  | 1.00 |  | 20 | 3.37 |  |  | 1.00 |  |
| **Day 35 pi** |  |  |  |  |  |  |  |  |  |  |  |  |
| Group | 1 | 0.20 | 0.20 | 1.30 | 0.06 | 0.225 | 1 | 0.29 | 0.29 | 1.94 | 0.09 | **0.033** |
| Residuals | 21 | 3.27 | 0.16 |  | 0.94 |  | 19 | 2.83 | 0.15 |  | 0.91 |  |
| Total | 22 | 3.47 |  |  | 1.00 |  | 20 | 3.12 |  |  | 1.00 |  |
| **Day 42 pi** |  |  |  |  |  |  |  |  |  |  |  |  |
| Group | 1 | 0.13 | 0.13 | 1.64 | 0.15 | 0.149 | 1 | 0.23 | 0.23 | 1.92 | 0.18 | **0.045** |
| Residuals | 9 | 0.74 | 0.08 |  | 0.85 |  | 9 | 1.09 | 0.12 |  | 0.82 |  |
| Total | 10 | 0.87 |  |  | 1.00 |  | 10 | 1.32 |  |  | 1.00 |  |
| **Day 49 pi** |  |  |  |  |  |  |  |  |  |  |  |  |
| Group | 1 | 0.17 | 0.17 | 1.36 | 0.13 | 0.225 | 1 | 0.35 | 0.35 | 2.42 | 0.21 | **0.003** |
| Residuals | 9 | 1.16 | 0.13 |  | 0.87 |  | 9 | 1.30 | 0.14 |  | 0.79 |  |
| Total | 10 | 1.33 |  |  | 1.00 |  | 10 | 1.66 |  |  | 1.00 |  |

Abbreviations: Df, degrees of freedom; MS, mean squares; pi, post infection; SS, sums of squares
