# Supplementary material for: Modulation of the porcine intestinal microbiota in the course of Ascaris suum infection
Source: Parasit Vectors. 2022 Nov 17;15:433. doi: 10.1186/s13071-022-05535-w (PMC9673396; doi:10.1186/s13071-022-05535-w)
Supplement: Supplementary file 3 — Additional file 3. Results of PERMANOVAs testing the effect of each A. suum experimental group vs the control group on the microbiota composition in ingesta samples from the ileum, caecum and colon on days 21, 35 and 49 p.i. Analyses were based on OTU-level Jensen–Shannon distance values. [file 13071_2022_5535_MOESM3_ESM.docx]

**Supplementary material 3:** Results of PERMANOVA analyses testing the effect of each *A. suum* experimental group vs. the control group on the microbiota composition in ingesta samples from the ileum, caecum and colon on days 21, 35 and 49 pi. Analyses were based on OTU-level Jensen-Shannon distance values.

|  |  | **Single-infection vs. control** | | | | | | | **Trickle-infection vs. control** | | | | | |
| --- | --- | --- | --- | --- | --- | --- | --- | --- | --- | --- | --- | --- | --- | --- |
| **Compartment (Day pi)** | **Term** | **Df** | **SS** | **MS** | **F** | **R^2^** | ***P*** | **Df** | | **SS** | **MS** | **F** | **R^2^** | ***P*** |
| **Ileum (21)** | Group | 1 | 0.17 | 0.17 | 5.59 | 0.41 | **0.031** | 1 | | 0.11 | 0.11 | 3.68 | 0.29 | 0.127 |
|  | Residuals | 8 | 0.24 | 0.03 | - | 0.59 | - | 9 | | 0.26 | 0.03 | - | 0.71 | - |
|  | Total | 9 | 0.41 | - | - | 1.00 | - | 10 | | 0.37 | - | - | 1.00 | - |
| **Ileum (35)** | Group | 1 | 0.07 | 0.07 | 4.12 | 0.34 | **0.006** | 1 | | 0.21 | 0.21 | 3.07 | 0.28 | 0.101 |
|  | Residuals | 8 | 0.14 | 0.02 | - | 0.66 | - | 8 | | 0.55 | 0.07 | - | 0.72 | - |
|  | Total | 9 | 0.22 | - | - | 1.00 | - | 9 | | 0.76 | - | - | 1.00 | - |
| **Ileum (49)** | Group | 1 | 0.08 | 0.08 | 2.49 | 0.22 | 0.173 | 1 | | 0.04 | 0.04 | 1.63 | 0.17 | 0.209 |
|  | Residuals | 9 | 0.29 | 0.03 | - | 0.78 | - | 8 | | 0.20 | 0.03 | - | 0.83 | - |
|  | Total | 10 | 0.37 | - | - | 1.00 | - | 9 | | 0.25 | - | - | 1.00 | - |
| **Caecum (21)** | Group | 1 | 0.20 | 0.20 | 2.90 | 0.27 | **0.044** | 1 | | 0.16 | 0.16 | 2.65 | 0.23 | 0.063 |
|  | Residuals | 8 | 0.55 | 0.07 | - | 0.73 | - | 9 | | 0.53 | 0.06 | - | 0.77 | - |
|  | Total | 9 | 0.75 | - | - | 1.00 | - | 10 | | 0.69 | - | - | 1.00 | - |
| **Caecum (35)** | Group | 1 | 0.30 | 0.30 | 5.79 | 0.39 | **0.002** | 1 | | 0.17 | 0.17 | 2.28 | 0.22 | 0.065 |
|  | Residuals | 9 | 0.47 | 0.05 | - | 0.61 | - | 8 | | 0.58 | 0.07 | - | 0.78 | - |
|  | Total | 10 | 0.77 | - | - | 1.00 | - | 9 | | 0.75 | - | - | 1.00 | - |
| **Caecum (49)** | Group | 1 | 0.01 | 0.01 | 0.05 | 0.01 | 0.994 | 1 | | 0.10 | 0.10 | 1.49 | 0.14 | 0.173 |
|  | Residuals | 10 | 0.60 | 0.06 | - | 0.99 | - | 9 | | 0.62 | 0.07 | - | 0.86 | - |
|  | Total | 11 | 0.60 | - | - | 1.00 | - | 10 | | 0.72 | - | - | 1.00 | - |
| **Colon (21)** | Group | 1 | 0.05 | 0.05 | 1.38 | 0.15 | 0.244 | 1 | | 0.08 | 0.08 | 2.12 | 0.21 | 0.085 |
|  | Residuals | 8 | 0.27 | 0.03 | - | 0.85 | - | 8 | | 0.29 | 0.04 | - | 0.79 | - |
|  | Total | 9 | 0.32 | - | - | 1.00 | - | 9 | | 0.37 | - | - | 1.00 | - |
| **Colon (35)** | Group | 1 | 0.04 | 0.04 | 1.0 | 0.09 | 0.443 | 1 | | 0.08 | 0.08 | 1.15 | 0.11 | 0.345 |
|  | Residuals | 10 | 0.41 | 0.04 | - | 0.91 | - | 9 | | 0.59 | 0.07 | - | 0.89 | - |
|  | Total | 11 | 0.45 | - | - | 1.00 | - | 10 | | 0.66 | - | - | 1.00 | - |
| **Colon (49)** | Group | 1 | 0.02 | 0.02 | 0.35 | 0.03 | 0.941 | 1 | | 0.10 | 0.10 | 2.20 | 0.20 | 0.085 |
|  | Residuals | 10 | 0.51 | 0.05 | - | 0.97 | - | 9 | | 0.40 | 0.04 | - | 0.80 | - |
|  | Total | 11 | 0.53 | - | - | 1.00 | - | 10 | | 0.50 | - | - | 1.00 | - |

Abbreviations: Df, degrees of freedom; MS, mean squares; SS, sum of squares
