# Supplementary material for: Modulation of the porcine intestinal microbiota in the course of Ascaris suum infection
Source: Parasit Vectors. 2022 Nov 17;15:433. doi: 10.1186/s13071-022-05535-w (PMC9673396; doi:10.1186/s13071-022-05535-w)
Supplement: Supplementary file 4 — Additional file 4. Heatmap of differentially abundant species in the (A) ileum and (B) caecum and colon of A. suum-infected pigs compared with a non-infected control group, as determined by DESeq2 analysis. Only differences with Benjamini–Hochberg-adjusted P-values ≤ 0.01 are shown as coloured tiles. [file 13071_2022_5535_MOESM4_ESM.pdf]

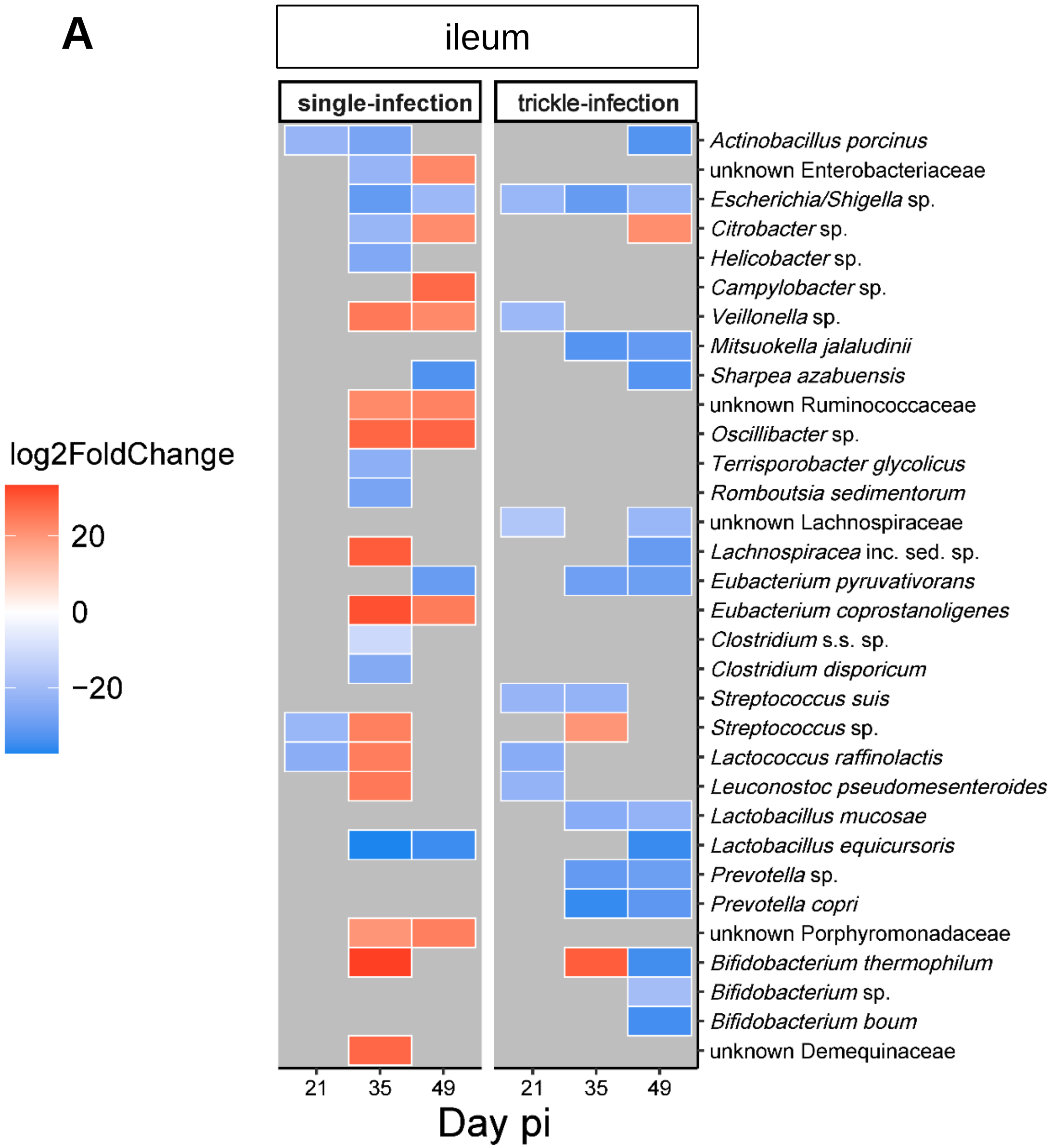

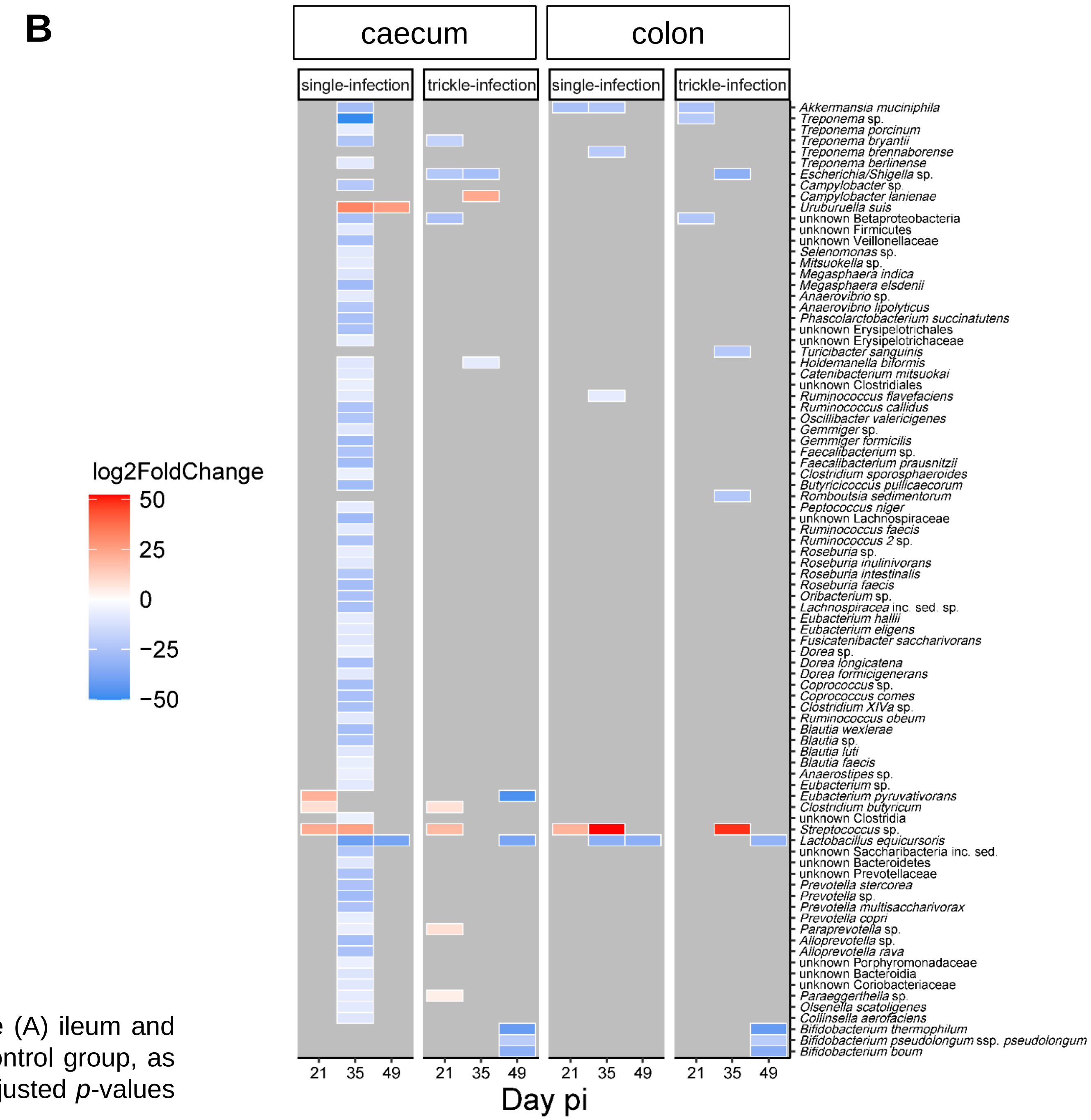

Supplementary material 4: Heatmap of differentially abundant species in the (A) ileum and (B) caecum and colon of *A. suum* infected pigs compared to a non-infected control group, as determined by DESeq2 analysis. Only differences with Benjamini-Hochberg adjusted *p*-values ≤ 0.01 are shown as coloured tiles.
